# Pooled Analysis of Non-Union, Re-Operation, Infection, and Approach Related Complications after Anterior Odontoid Screw Fixation

**DOI:** 10.1371/journal.pone.0103065

**Published:** 2014-07-24

**Authors:** Nai-Feng Tian, Xu-Qi Hu, Li-Jun Wu, Xin-Lei Wu, Yao-Sen Wu, Xiao-Lei Zhang, Xiang-Yang Wang, Yong-Long Chi, Fang-Min Mao

**Affiliations:** 1 Department of Orthopaedic Surgery, Second Affiliated Hospital of Wenzhou Medical University, Wenzhou, Zhejiang, China; 2 Institute of Digitized Medicine, Wenzhou Medical University, Wenzhou, Zhejiang, China; 3 Department of Orthopaedics, Second Affiliated Hospital, School of Medicine, Zhejiang University, Hangzhou, Zhejiang, China; 4 Center for Stem Cells and Tissue Engineering, School of Medicine, Zhejiang University, Hangzhou, Zhejiang, China; Toronto Western Hospital, Canada

## Abstract

**Background:**

Anterior odontoid screw fixation (AOSF) has been one of the most popular treatments for odontoid fractures. However, the true efficacy of AOSF remains unclear. In this study, we aimed to provide the pooled rates of non-union, reoperation, infection, and approach related complications after AOSF for odontoid fractures.

**Methods:**

We searched studies that discussed complications after AOSF for type II or type III odontoid fractures. A proportion meta-analysis was done and potential sources of heterogeneity were explored by meta-regression analysis.

**Results:**

Of 972 references initially identified, 63 were eligible for inclusion. 54 studies provided data regarding non-union. The pooled non-union rate was 10% (95% CI: 7%–3%). 48 citations provided re-operation information with a pooled proportion of 5% (95% CI: 3%–7%). Infection was described in 20 studies with an overall rate of 0.2% (95% CI: 0%–1.2%). The main approach related complication is postoperative dysphagia with a pooled rate of 10% (95% CI: 4%–17%). Proportions for the other approach related complications such as postoperative hoarseness (1.2%, 95% CI: 0%–3.7%), esophageal/retropharyngeal injury (0%, 95% CI: 0%–1.1%), wound hematomas (0.2%, 95% CI: 0%–1.8%), and spinal cord injury (0%, 95% CI: 0%–0.2%) were very low. Significant heterogeneities were detected when we combined the rates of non-union, re-operation, and dysphagia. Multivariate meta-regression analysis showed that old age was significantly predictive of non-union. Subgroup comparisons showed significant higher non-union rates in age ≥70 than that in age ≤40 and in age 40 to <50. Meta-regression analysis did not reveal any examined variables influencing the re-operation rate. Meta-regression analysis showed age had a significant effect on the dysphagia rate.

**Conclusions/Significances:**

This study summarized the rates of non-union, reoperation, infection, and approach related complications after AOSF for odontoid factures. Elderly patients were more likely to experience non-union and dysphagia.

## Introduction

Odontoid fractures account for 10%–15% of all cervical spine fractures [1]. Despite of the frequency of odontoid fractures, its management remains controversial and ranges from conservative treatment to surgical intervention [2]–[7]. Conservative treatment consists of skull traction, cervical collar, brace, and halo vest. However, such methods are unpopular for unstable odontoid fractures (type II and shallow type III based on the classification of Anderson and D'lonzo [8]) because of the high non-union rate [2], [3]. Moreover, they are often poorly tolerated in the elderly and in the multiply injured patients [6]. Posterior C1–C2 fusion has been advocated as it significantly increases the fusion rate [9], [10]. Nevertheless, this technique is associated with extensive surgical exposure, autogenous bone harvest, and compromise of the cervical movement [2]–[6]. Anterior odontoid screw fixation (AOSF) has been one of the surgical treatments for unstable odontoid fractures since it was independently introduced by Bohler and Nakanishi [11], [12]. This technique seems an ideal treatment as it preserves C1–C2 movement and obviates bone graft harvest.

To date, many clinical studies have evaluated the effectiveness of AOSF for odontoid fractures [12]–[77]. Nevertheless, clinicians may be confused about the true efficacy of this technique because of the wide variability of these reports. Previous reviews were mostly narrative or were investigations that did not weight the results of the single studies according to the number of participants [2], [4]–[6]. Therefore, it is important to combine the results from different studies for clinical reference. In this study, we aimed to provide pooled rates of non-union, re-operation, infection, and approach related complications after surgical treatment of unstable odontoid fractures using AOSF. Furthermore, we tried to explore potential factors that affected these outcomes.

## Materials and Methods

### Search strategy and inclusion criteria

A computerized systematic search was conducted up to August 2013 using MEDLINE database. We screened all fields by the term “odontoid fracture” or “odontoid screw” or “odontoid fixation”. Articles were limited to those published in English. We also searched the reference lists and relevant journals by hand. This meta-analysis was performed in accordance with the preferred reporting items for systematic reviews and meta-analyses (PRISMA) guidelines (when appropriate) (Checklist S1).

We included studies which were carried out on humans. The studies that discussed fusion results, and/or re-operations, and/or infections, and/or approach related complications after odontoid screw fixation for type II or type III odontoid fractures were selected. We also required a minimum sample size of five to ensure quality and comparability of data. Biomechanical studies, cadaveric studies, animal studies, case report, duplications, and review articles were excluded. Clinical studies with inadequate information or incomplete data were also excluded. We carefully reviewed the department/institute of each potential eligible study to screen whether there were any papers from the same surgical team. For papers that had overlapping patients determined through the overlapping research time, we only included the paper with the largest sample size for analysis.

### Data extraction

Two investigators reviewed all identified articles to determine if an individual study was eligible for inclusion. Data from each study was extracted using a standardized form. Disagreements on eligibility and data between reviewers were resolved by consensus with the third reviewer. Studies were categorized into levels of evidence according to those published in the Journal of Bone and Joint Surgery (American) [78].

Data extracted consisted of study year, country, level of evidence, patients' mean age, sex proportion, mean follow-up duration, classification of the odontoid fracture, number of patients, number of non-unions, number of re-operations, number of infections, and number of approach related complications. Fusion status should be assessed according to radiological (static or dynamic) and/or computed tomography (CT) examinations. Because the fusion criteria might be different among the included studies, we used a universal definition of fusion for data extraction. Criteria for fusion success included formation of trabecular and cortex bony bridges through the fracture site, absence of sclerotic borders adjacent to the fracture site, and absence of movement of the fracture site confirmed on radiographs and/or CT scan. Radiolucent cleft, clear fracture line, fibrous union, or any movement at the fracture site were considered as non-union. Re-operation represented secondary surgical intervention for any reason after odontoid screw fixation. Infections indicated only those located at the surgical site including both superficial and deep ones. We extracted data on five types of approach related complications including postoperative dysphagia, postoperative hoarseness, esophageal /retropharyngeal injury, wound hematomas, and spinal cord injury. In this meta-analysis, data regarding the study characteristics and the outcomes of interest were extracted based on the average value of each study. For studies with overlapping patients, only the one with the largest sample size was entered into meta-analysis.

### Statistical analysis

Meta-analyses were performed to pool the rates of non-union, re-operation, infection, and approach related complications. A Freeman-Tukey Double arcsine transformation was implemented to calculate the overall proportion. A test of heterogeneity was carried out, and cut-off p value of 0.1 was established as a threshold of homogeneity. Pooled estimates and 95% confidence intervals (CIs) were summarized by forest plots. Fixed-effect models were applied unless statistical heterogeneity was significant, in which case random-effect models were used. We further investigated potential sources of heterogeneity by arranging groups of studies according to relevant characteristics (year of publication, level of evidence, patients' mean age, sex, follow-up duration, fracture type, and study sample size) and by meta-regression analysis. Factors were examined both individually and in multiple-variable models. To avoid model instability, only factors that showed significant effects individually were enrolled into a multiple regression model. Publication bias was assessed using Egger test. All analyses were done in the statistical software R 3.0.1.

## Results

**Figure 1:**
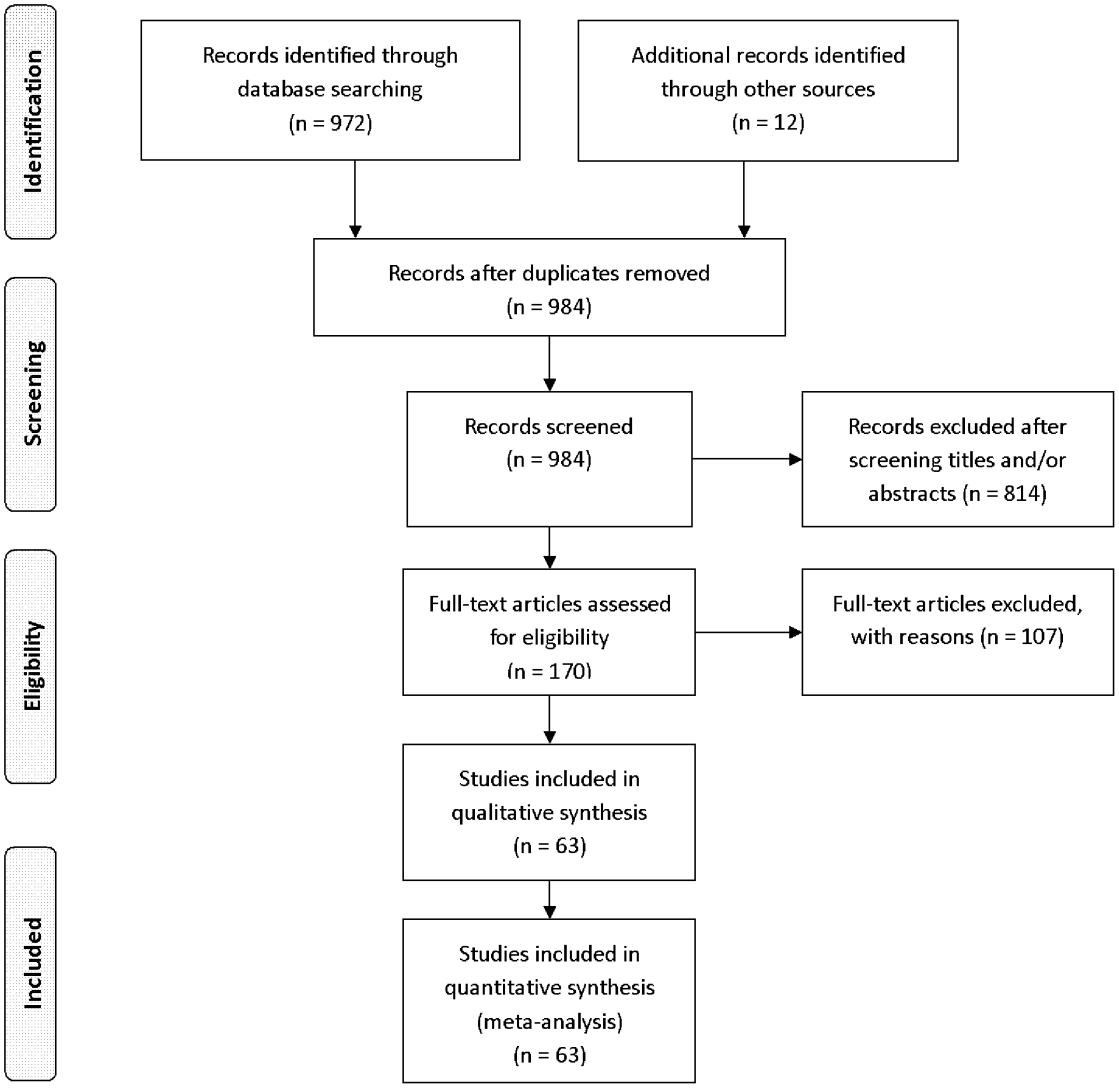
Selection of relevant publications, reasons for exclusion.

In the initial screening, 972 potential studies were selected according to the search strategy. Hand-searching resulted in 12 additional papers. After reviewing titles and abstracts, 814 papers were found to be unrelated to the current topic. Of the remaining 170 papers, the full texts were read and 104 publications which did not meet eligibility criteria were excluded. Further three papers [71], [73], [76] were excluded due to potential overlapping patients. Consequently, sixty three papers met all inclusion criteria and were selected (Figure 1) [12]–[70], [72], [74], [75], [77]. Based on the level of evidence, there were 1 level II, 17 level III, and 45 level IV studies. The mean age ranged from 35 to 85.4 years. We divided the studies into five age subgroups (age ≤40, age 40 to <50, age 50 to <60, age 60 to <70, and age ≥70). The male to female ratio was 1.74. The follow-up duration ranged from 1.5 months to 9 years. 88.9% of the injuries were type II dens fractures according to Anderson and D'Alonzo's classification [8]. The characteristics of selected studies were summarized in Table S1 and Table S2.

**Figure 2:**
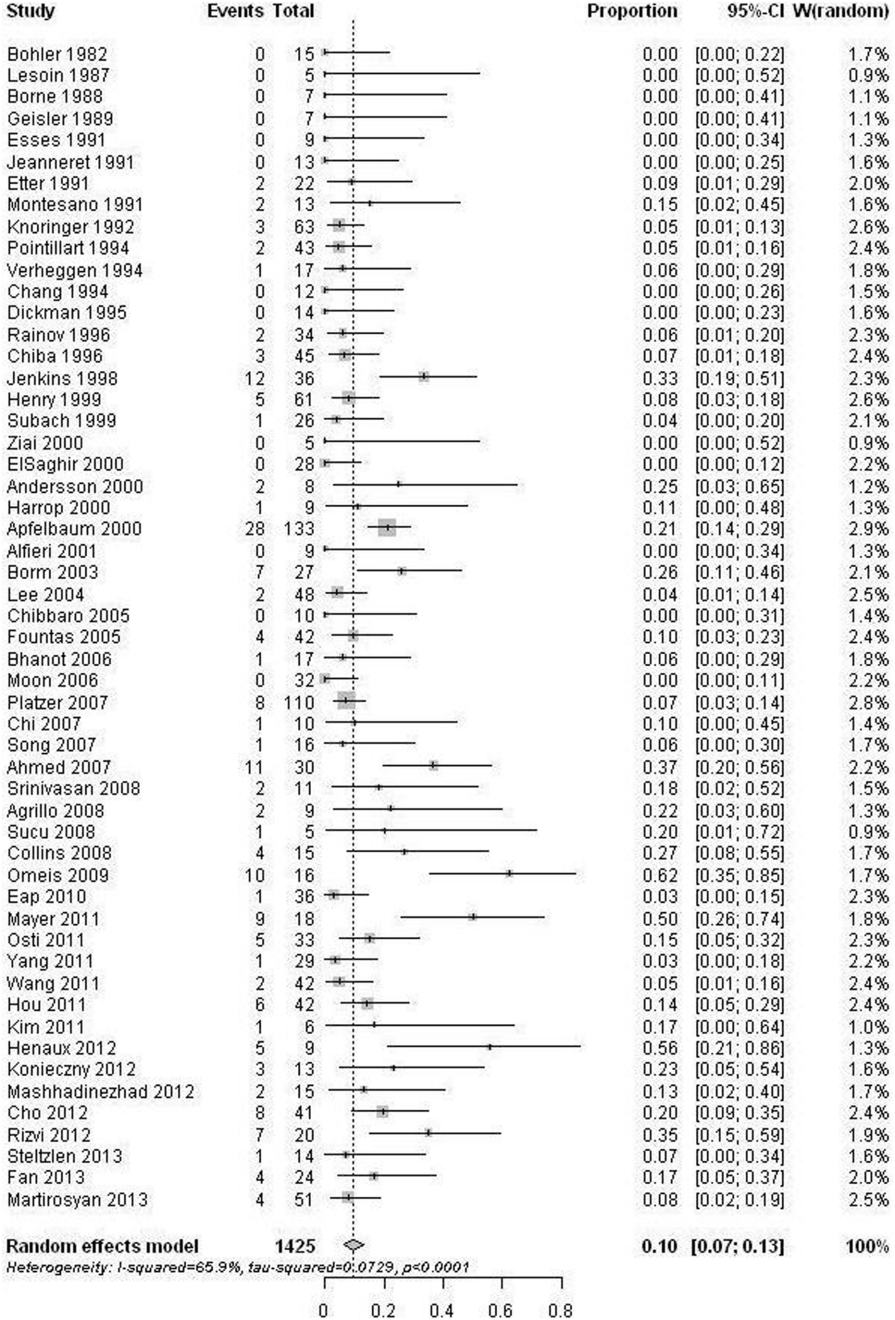
Forest plots showing the non-union rates (boxes) with 95% confidence of intervals (CIs; bars).

**Figure 3:**
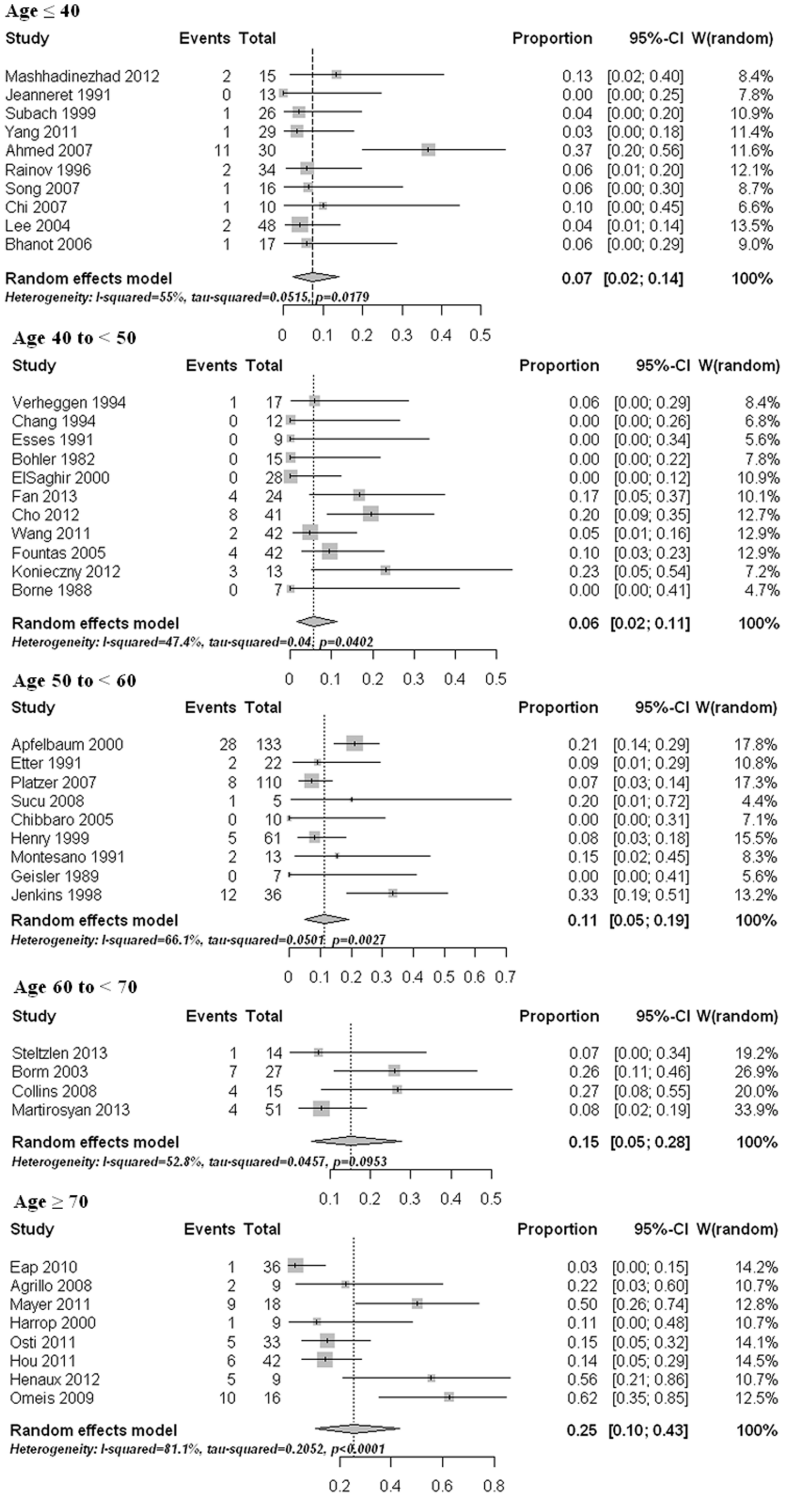
Forest plots showing the non-union rates (boxes) with 95% confidence of intervals (CIs; bars) in different age groups.

54 studies [12]–[20], [22]–[29], [31]–[51], [53]–[60], [62], [63], [65]–[70] that reported fusion results were pooled. Six studies [21], [52], [61], [72], [74], [75] were excluded from data synthesizing because of potential patients overlapping. Imaging methods used for fusion assessment included radiograph (static and/or dynamic) and CT scan. Radiograph was used in fifty two studies. CT scan was used in twenty six papers. Twenty five studies used both methods. In selected studies, a total of 1425 patients were evaluated. The non-union rates ranged from 0% to 62%. The pooled estimate for all studies was 10% (95% CI: 7%–13%). However, the estimate was associated with substantial heterogeneity (p<0.001) (Figure 2). We observed that the pooled non-union rate based on CT scan (12%, 95% CI: 7%–17%) was higher than that based on only X-rays (8%, 95% CI: 4%–13%). Nevertheless, the difference was statistically insignificant (p = 0.234) after univariate meta-regression analysis. Therefore, we combine both image modalities into one database. Univariate meta-regression analysis showed that old age (p = 0.002), less than one year follow-up (p = 0.017), and publication after 2000 (p = 0.012) were significantly predictive of non-union. The non-union rate increased with age, as estimates in the five age groups were 7%, 6%, 11%, 15%, and 25%, respectively (Figure 3). Subgroup comparisons showed that the non-union rate in age ≥70 was significant higher than that in age ≤40 (p = 0.015) and in age 40 to <50 (p = 0.015). After multivariate meta-regression analysis, only age (p = 0.016) remained significant. No significant publication bias was detected (p = 0.699).

**Figure 4:**
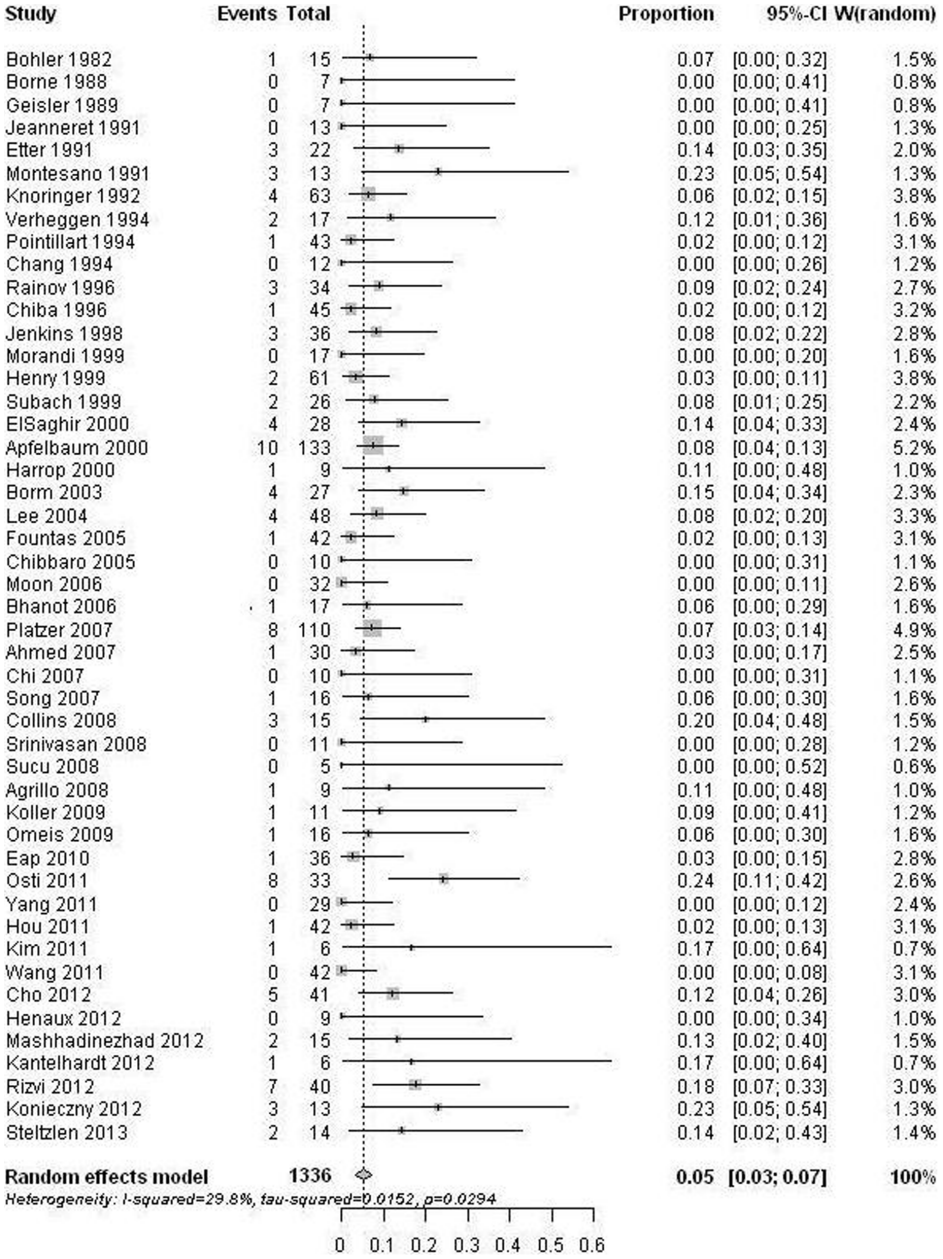
Forest plots showing the re-operation rates (boxes) with 95% confidence of intervals (CIs; bars).

48 citations [12], [14], [15], [17]–[20], [22]–[24], [26]–[31], [33]–[35], [38]–[56], [58]–[60], [62]–[67], [69] provided re-operation information following odontoid screw fixation. Reasons for re-operation included screw loosening/pullout/cut-out/mal-position, fracture re-dislocation, unstable non-union, hematoma, and so on. The re-operation rates ranged from 0% to 24%. The random-effect pooled proportion was 5% (95% CI: 3%–7%) with pronounced heterogeneity (p = 0.029) (Figure 4). Meta-regression analysis revealed that none of the examined variables (year of publication, age, gender, follow-up duration, fracture type, or sample size) significantly influenced the re-operation rate. Egger test for publication bias showed no significant evidence for bias (p = 0.343).

**Figure 5:**
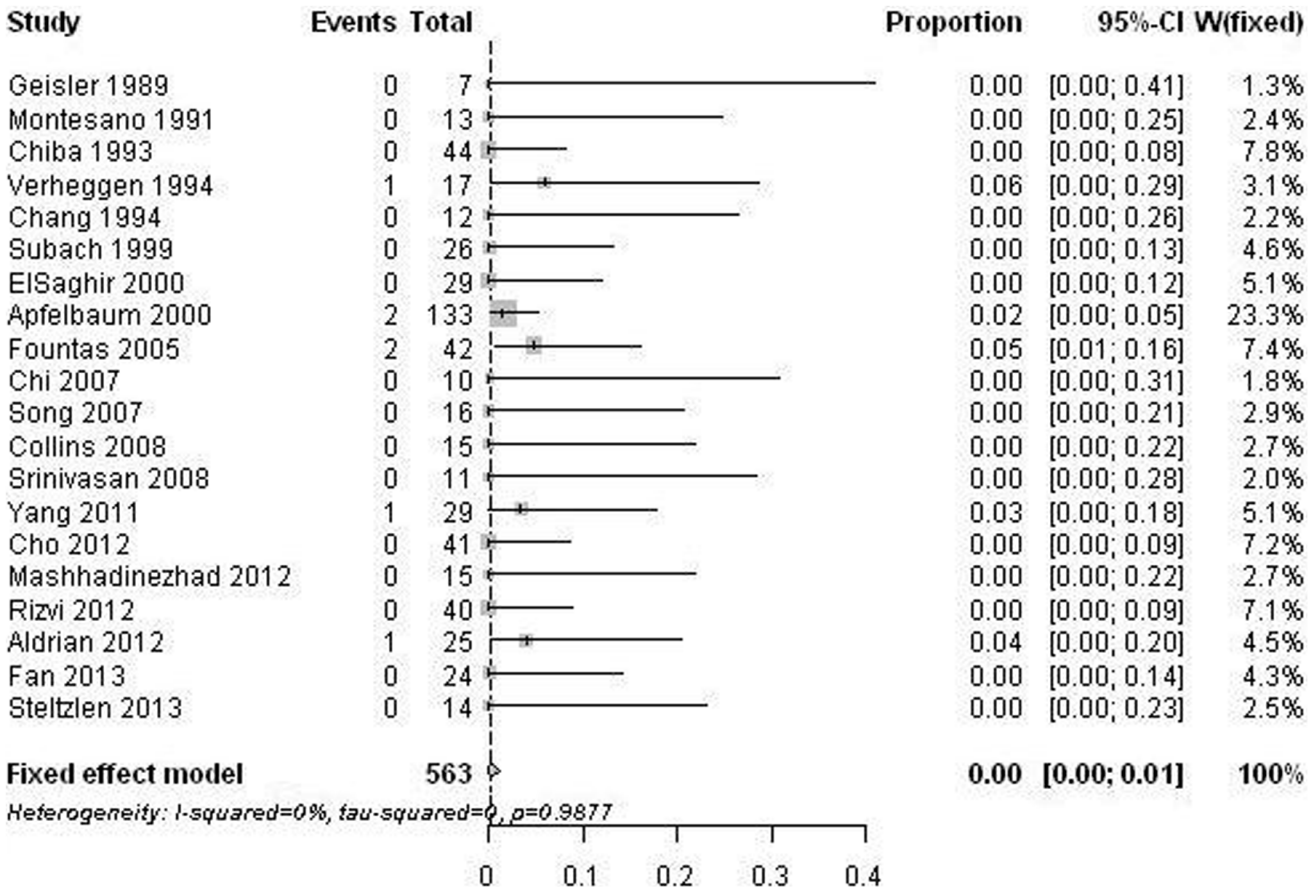
Forest plots showing the infection rates (boxes) with 95% confidence of intervals (CIs; bars).

Surgical site infection was assessed in 20 studies [15], [19], [21], [22], [24], [31], [33], [34], [41], [45], [47], [49], [50], [60]–[62], [66], [67], [69], [70] with 563 surgeries. The reported infection rate was low, with estimates varied from 0% to 6%. The overall infection rate of all included studies was 0.2% (95% CI: 0%–1.2%) (Figure 5). As there was no substantial significant heterogeneity, further meta-regression analysis was not carried out. There was no significant publication bias (p = 0.549).

**Figure 6:**
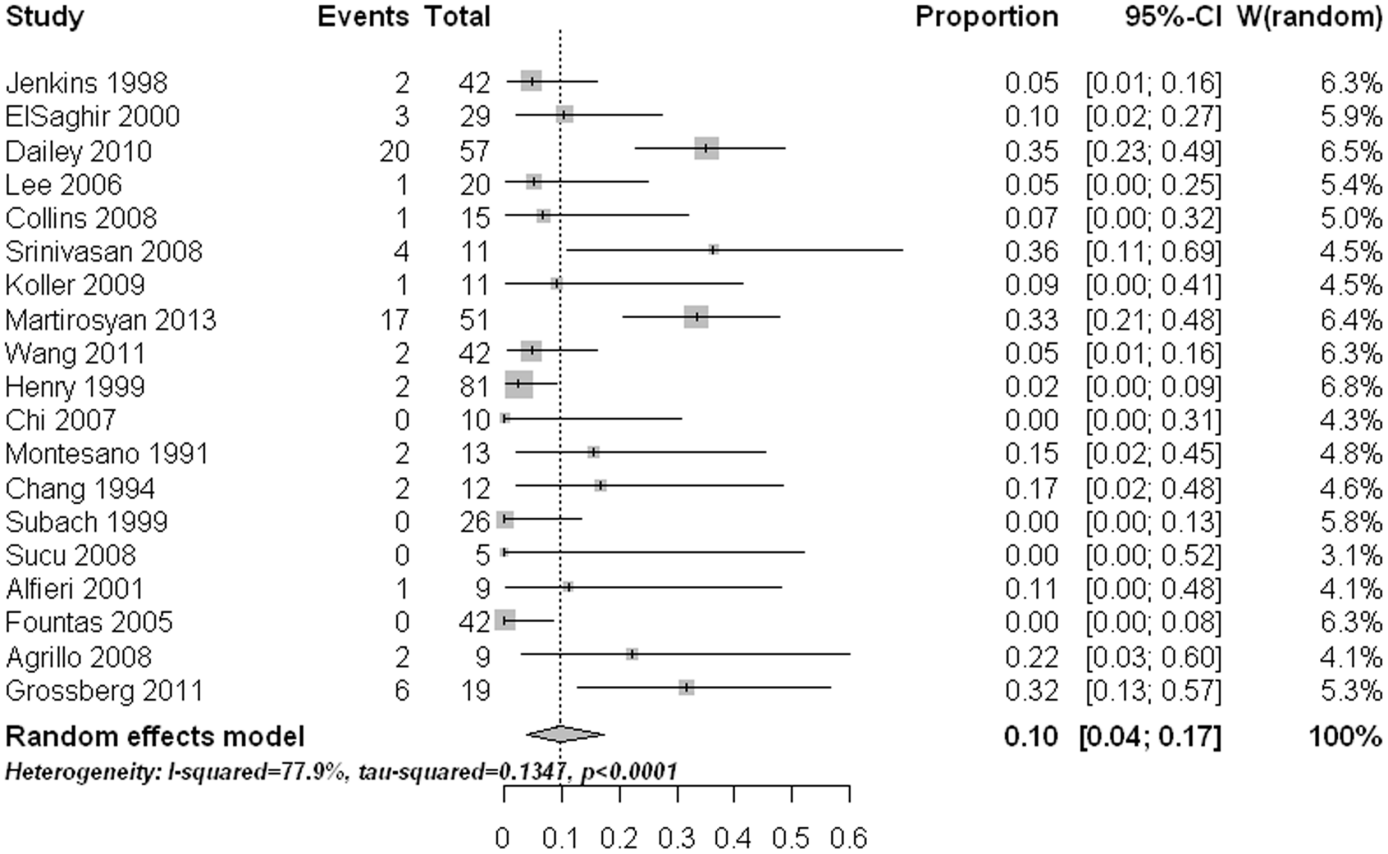
Forest plots showing the rates of dysphagia (boxes) with 95% confidence of intervals (CIs; bars).

**Figure 7:**
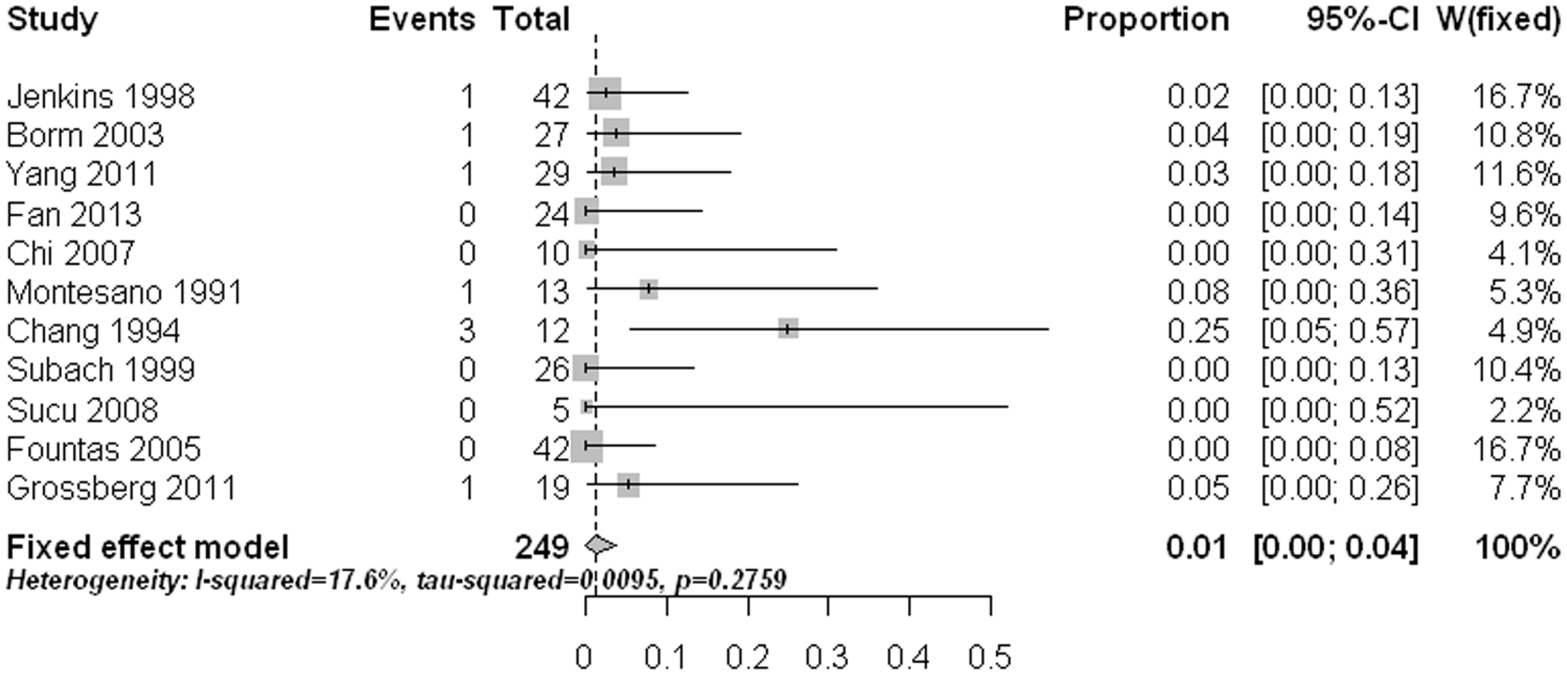
Forest plots showing the rates of hoarseness (boxes) with 95% confidence of intervals (CIs; bars).

**Figure 8:**
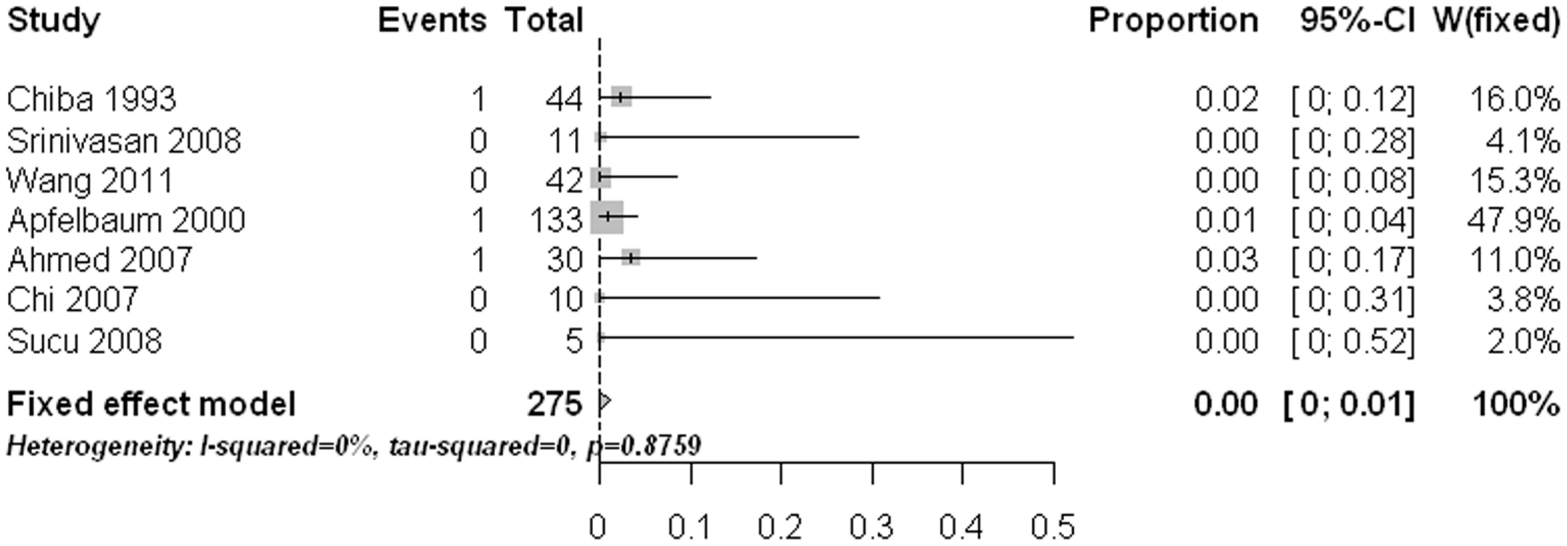
Forest plots showing the rates of esophageal /retropharyngeal injury (boxes) with 95% confidence of intervals (CIs; bars).

**Figure 9:**
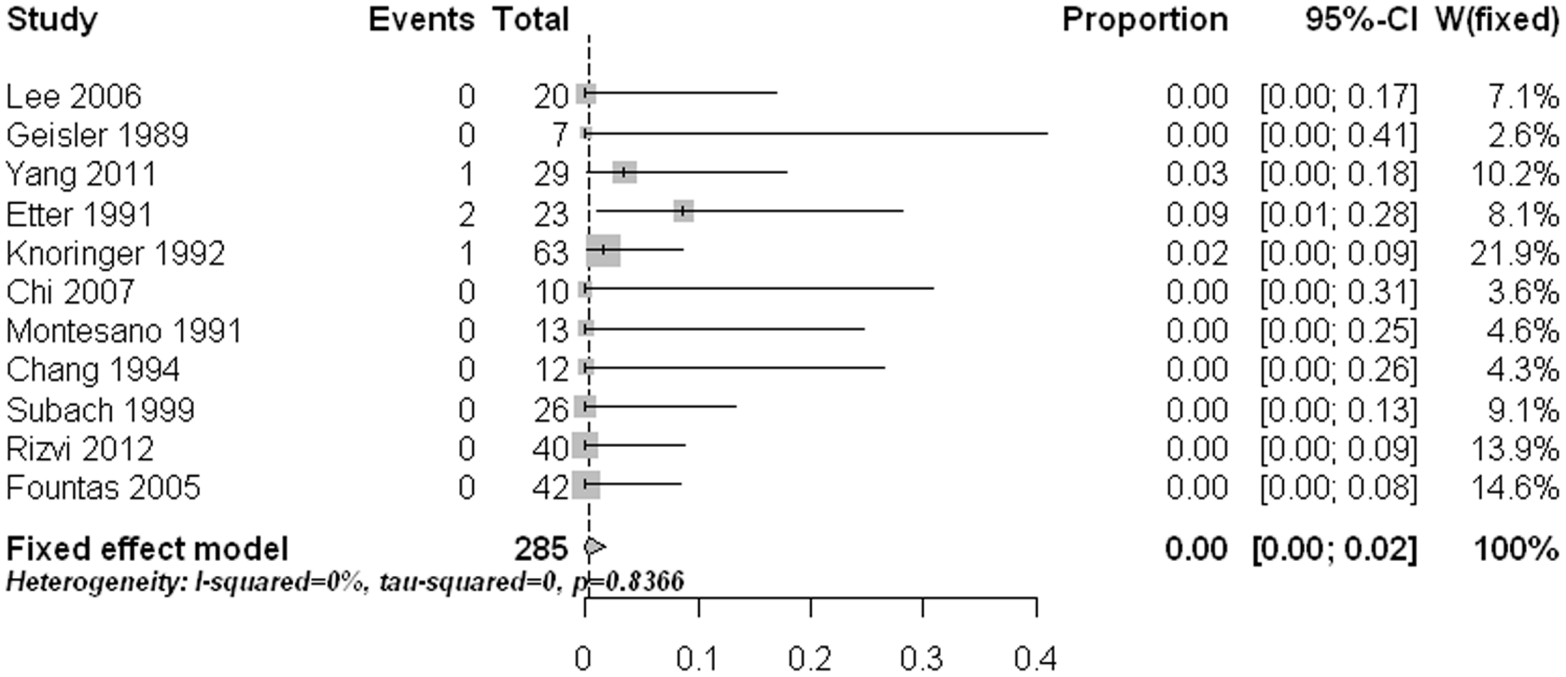
Forest plots showing the rates of wound hematomas (boxes) with 95% confidence of intervals (CIs; bars).

**Figure 10:**
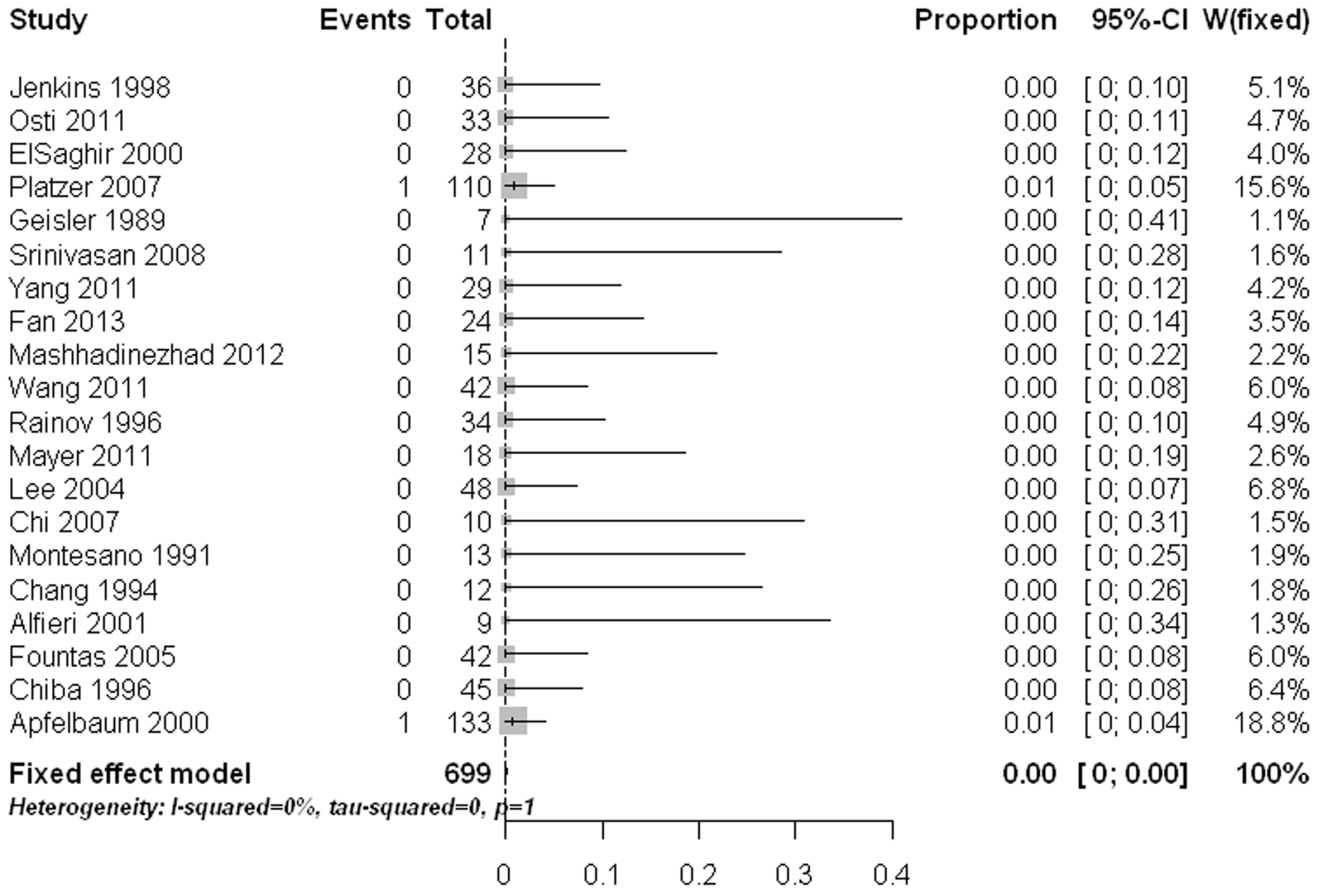
Forest plots showing the rates of spinal cord injury (boxes) with 95% confidence of intervals (CIs; bars).

The main approach related complication was postoperative dysphagia. The pooled rate was 10% (95% CI: 4%–17%) with statistically significant heterogeneity (Figure 6). Meta-regression analysis revealed that age had a significant effect on the estimate (p<0.0001). Subgroup comparisons indicated that the two old age groups (age 60 to <70 yrs and age ≥70 yrs) had significant higher dysphagia rates than those in the other three age groups (age ≤40, age 40 to <50, and age 50 to <60) (p<0.05). Pooled proportions for the other approach related complications like postoperative hoarseness (1.2%, 95% CI: 0%–3.7%) (Figure 7), esophageal /retropharyngeal injury (0%, 95% CI: 0%–1.1%) (Figure 8), wound hematomas (0.2%, 95% CI: 0%–1.8%) (Figure 9), and spinal cord injury (0%, 95% CI: 0%–0.2%) (Figure 10) were very low. No significant publication bias was detected (p>0.1)

## Discussion

We conducted this study to provide a better understanding of the frequency of non-union, infection, re-operation, and approach related complications after anterior screw fixation for type II and type III odontoid fractures. Non-union can be one of the most important outcomes, because it may lead to spinal cord injury due to atlantoaxial instability. Pooled analysis from our study showed that the non-union rate after AOSF was 10%. It seemed that the fusion rate of AOSF (90%) was better than that of the conservative treatment (60%–80%) [3], and was comparable to that of the posterior fixation (89%–100%) [5]. Therefore, AOSF might be a good choice for type II and type III odontoid fractures in selected patients. This study revealed that the re-operation rate was 5% after AOSF. The reasons for re-operation included non-union, screw failure, fracture re-dislocation, and occasionally hematoma. Since non-union accounted for fifty percent of the cases undergoing re-operation, obtaining bony fusion becomes the first priority in AOSF. Not all of the non-unions underwent second surgical interventions, because some of them (fibrous unions) were radiologically stable. For these cases, long term follow up was still essential. The infection rate in surgical site was very low with only seven cases identified during our review [24], [33], [41], [60], [61]. The pooled estimate was 0.2% without significant heterogeneity among the studies. All infection cases were superficial and were resolved without sequelae.

Our study revealed that age had a significant impact on the non-union rate. The non-union rate in patients younger than 50 years was 6%. Therefore, AOSF seems to be a good choice for young patients. Although the non-union rate reached 11% to 25% in patients aged 50 years or older, this rate was still acceptable as the non-union rate of conservative treatment for the elderly patients was very high (60% in Nourbakhsh' review [3], and 56%–72% in Huybregts' review [7]). Subgroup comparisons showed that age ≥70 had a significant higher non-union rate than the young had. Our findings were consistent with those reported by Platzer et al [46]. They observed that patients older than 65 years had a significantly higher non-union rate of 12% compared with that of 4% in younger individuals [46]. However, two other observational studies reported that age was not associated with fusion failure [38], [62]. It was generally agreed that old patients had a higher chance to experience osteoporosis and diminished bone quality which might have an important effect on the fusion outcome. As none of the studies directly assessed osteoporosis of surgical patients, the bone quality information in different age groups was not clear. This could be one reason to explain the controversial results from different studies. Therefore, it is important for further studies to clarify the relationships among age, osteoporosis, and fusion outcomes after AOSF. Since elderly patients were more likely to experience non-unions, measures should be adopted to enhance the bony fusion in this population. Dailey et al [75] retrospectively analyzed the efficacy of AOSF in a group of patients with age over 70. They observed a significantly higher stabilization rate of 96% in patients when 2 screws were placed, compared with that of 56% in patients with only one screw used. However, in another group of relatively young patients, the difference became statistically insignificant [28]. Younger patients have better bone quality which could provide more stability at the surgical site. Thus, placing one screw may be sufficient. Nevertheless, the elderly patients might benefit from an additional screw which added rotational stability in the osteopenic bone [75].

Postoperative dysphagia was the main approach related complication after AOSF with pooled estimate of 10%, followed by postoperative hoarseness (1.2%). Esophageal /retropharyngeal injury, wound hematomas, and spinal cord injury were rare approach related complications. Noteworthy was that age also had a significant effect on postoperative dysphagia rate. Dysphagia is a known complication of anterior cervical spine surgery. A recent systematic review showed that female gender, advanced age, multilevel surgery, longer operating time and severe pre-operative neck pain may increase the risk of postoperative dysphagia after cervical spine surgery [79]. During our review, there was no study directly comparing the dysphagia rates among different age groups. Through this meta-analysis, we observed that age ≥60 had a significant higher dysphagia rate than the age <60 had. The possible reason for this fact was that the elderly patient's esophagus was less tolerant to retraction due to fibrosis [75]. Considering the relatively high dysphagia rate in the elderly after AOSF, strategies, such as using of perioperative methylprednisolone, monitoring of endotracheal tube cuff pressure, and preoperative tracheal/esophageal traction exercise, may be employed to reduce the risk of this complication [79].

There are some limitations existing in this study. First, this meta-analysis only focused on the rates of non-union, re-operation, infection, and approach related complications. We did not pool other outcomes like functional results and patient satisfactory outcome because they were not always reported or were reported in various forms. Even the outcomes we combined were not always available. Second, during the extraction of fusion data, we found the fusion status was assessed using different imaging modalities and non-union was defined according to different standards. Thus, pooling of relevant data might lead to bias even though we had predefined unified criteria for non-union. Third, extensive and significant heterogeneities were detected when we combined the rates of non-union, re-operation, and dysphagia. We had explored the heterogeneity through meta-regression analysis according to several study characteristics, but we only found age had a significant effect on the non-union and dysphagia rate. After subgroup analysis, we still observed heterogeneity in each age group, which meant there were potential other factors influencing the two outcomes. For re-operation, we failed to find potential factors which could explain the heterogeneity. The factors we analyzed represented the average value of each study, which could limit the exploration of the heterogeneity. Moreover, the heterogeneity might also be ascribed to various factors, such as other patient characteristics, fracture subtypes, and surgical techniques used. Lastly, the level of evidence of our analysis is low as none of the enrolled studies were randomized controlled trials. Despite these weaknesses, our study obtains some clinical significance since we pooled estimates based on a relatively large sample. This study provides a quantitative description of the frequencies of non-union, re-operation, infection, and approach related complications after AOSF for odontoid fractures. These data can be helpful in making informed surgical decisions. Further studies may be necessary to pool the functional outcomes of using this technique and to determine the factors affecting the efficacy.

## Supporting Information

Table S1 **Characteristics of the studies included for analyzing non-union, re-operation, and infection.**
(DOC)

Table S2 **Characteristics of the studies included for analyzing approach related complications.**
(DOC)

Checklist S1 **PRISMA Checklist.**
(DOC)
