# Supplementary material for: Pooled Analysis of Non-Union, Re-Operation, Infection, and Approach Related Complications after Anterior Odontoid Screw Fixation
Source: PLoS One. 2014 Jul 24;9(7):e103065. doi: 10.1371/journal.pone.0103065 (PMC4109995; doi:10.1371/journal.pone.0103065)
Supplement: Table S1 — Characteristics of the studies included for analyzing non-union, re-operation, and infection. (DOC) [file pone.0103065.s001.doc]

**Table S1.** Characteristics of the studies included for analyzing non-union, re-operation, and infection.

| **Study** | **Country** | **LE** | **Age**  **(year)** | **Male%** | **Follow-up**  **(month)** | **Type Ⅱ%** | **Sample**  **(n)** | **NU**  **(n)** | **RO**  **(n)** | **I**  **(n)** |
| --- | --- | --- | --- | --- | --- | --- | --- | --- | --- | --- |
| Bohler 1982 | Austria | IV | 44.8 | 73.3 | >1.5 | 46.7 | 15 | 0 | 1 | NA |
| Lesoin 1987 | France | IV | NA | NA | NA | NA | 5 | 0 | NA | NA |
| Borne 1988 | France | IV | 48.9 | 85.7 | NA | NA | 7 | 0 | 0 | NA |
| Geisler 1989 | USA | IV | 58 | 66.7 | >6 | 100 | 7 | 0 | 0 | 0 |
| Jeanneret 1991 | Switzerland | IV | 34 | 75 | 40.4 | 42.9 | 13 | 0 | 0 | NA |
| Etter 1991 | Switzerland | IV | 53.8 | 72.7 | >12 | 81.8 | 22 | 2 | 3 | NA |
| Montesano 1991 | USA | IV | 57 | 71.4 | 24 | 100 | 13 | 2 | 3 | 0 |
| Esses 1991 | Canada | IV | 43 | NA | >12 | 80 | 9 | 0 | NA | NA |
| Knoringer 1992 | Germany | IV | NA | NA | NA | NA | 63 | 3 | 4 | NA |
| Chiba 1993 | Japan | IV | 38 | 81.2 | 22 | 77.8 | 44 | 3 | NA | 0 |
| Verheggen 1994 | Germany | IV | 40.6 | 94.4 | NA | 88.9 | 17 | 1 | 2 | 1 |
| Pointillart 1994 | France | IV | NA | NA | >6 | NA | 43 | 2 | 1 | NA |
| Chang 1994 | Taiwan | III | 42 | 83.3 | >12 | 100 | 12 | 0 | 0 | 0 |
| Dickman 1995 | USA | IV | NA | NA | >12 | 100 | 14 | 0 | NA | NA |
| Rainov 1996 | Germany | IV | 36 | 65.7 | >6 | 91.4 | 34 | 2 | 3 | NA |
| Chiba 1996 | Japan | IV | NA | NA | >24 | 78.3 | 45 | 3 | 1 | NA |
| Jenkins 1998 | USA | III | 59 | 57 | 8.5 | 100 | 36 | 12 | 3 | NA |
| Morandi 1999 | France | IV | 51.8 | 58.8 | >1.5 | NA | 17 | NA | 0 | NA |
| Henry 1999 | France | IV | 57 | 59.3 | 16.6 | 35.8 | 61 | 5 | 2 | NA |
| Subach 1999 | USA | IV | 35 | 61.5 | 30 | 100 | 26 | 1 | 2 | 0 |
| Ziai 2000 | Canada | III | NA | NA | 6 | 100 | 5 | 0 | NA | NA |
| ElSaghir 2000 | Germany | IV | 45 | 53 | 26 | 100 | 28 | 0 | 4 | 0 (29)^1^ |
| Apfelbaum 2000 | USA | III | 50.1 | 67 | 18.2 | 93.9 | 133 | 28 | 10 | 2 |
| Harrop 2000 | USA | IV | 79.6 | 44.4 | 8.6 | 100 | 9 | 1 | 1 | NA |
| Andersson 2000 | Sweden | III | >66 | 63.6 | >24 | 90.9 | 8 | 2 | NA | NA |
| Alfieri 2001 | Italy | IV | >24 | 66.7 | NA | 100 | 9 | 0 | NA | NA |
| Borm 2003 | Germany | III | 66.8 | 59.3 | 16.6 | 100 | 27 | 7 | 4 | NA |
| Lee 2004 | Taiwan | IV | 37.2 | 77.1 | 14.6 | 81.3 | 48 | 2 | 4 | NA |
| Fountas 2005 | USA | IV | 47.7 | 64 | >24 | NA | 42 | 4 | 1 | 2 |
| Chibbaro 2005 | Italy | IV | 55 | 80 | 16 | 100 | 10 | 0 | 0 | NA |
| Moon 2006 | Korea | III | >23 | NA | >12 | 93.8 | 32 | 0 | 0 | NA |
| Bhanot 2006 | India | IV | 38.2 | 76.5 | 38.4 | 100 | 17 | 1 | 1 | NA |
| Platzer 2007 | Austria | III | 54 | 46.4 | >24 | 100 | 110 | 8 | 8 | NA |
| Ahmed 2007 | Saudi | IV | 35 | 83.3 | 10 | 100 | 30 | 11 | 1 | NA |
| Chi 2007 | China | IV | 37.2 | 60 | 15.7 | 60 | 10 | 1 | 0 | 0 |
| Song 2007 | Korea | IV | 36.3 | 81.3 | 41.5 | 75 | 16 | 1 | 1 | 0 |
| Collins 2008 | UK | IV | 68.9 | 73.3 | 18.3 | 100 | 15 | 4 | 3 | 0 |
| Srinivasan 2008 | India | IV | >17 | NA | >2 | 100 | 11 | 2 | 0 | 0 |
| Sucu 2008 | Turkey | IV | 54.8 | 60 | 15.7 | 60 | 5 | 1 | 0 | NA |
| Agrillo 2008 | Italy | IV | 73 | 66.7 | 8 | 100 | 9 | 2 | 1 | NA |
| Koller 2009 | Austria | IV | 57.9 | 90.9 | 72.8 | 90.9 | 11 | 0 | 1 | NA |
| Omeis 2009 | USA | IV | >70 | NA | 9 | 100 | 16 | 10 | 1 | NA |
| Eap 2010 | France | IV | 70.3 | 41.7 | 36 | 72.2 | 36 | 1 | 1 | NA |
| Osti 2011 | Austria | III | 79.6 | 55 | 67.2 | 100 | 33 | 5 | 8 | NA |
| Yang 2011 | China | III | 35 | 69 | 13.5 | 82.8 | 29 | 1 | 0 | 1 |
| Hou 2011 | China | IV | 80.6 | 55.8 | 21.3 | 100 | 42 | 6 | 1 | NA |
| Kim 2011 | Korea | III | NA | NA | >12 | NA | 6 | 1 | 1 | NA |
| Wang 2011 | China | II | 47.1 | 61.9 | 25.1 | 82.2 | 42 | 2 | 0 | NA |
| Mayer 2011 | Austria | III | 78.1 | 72.2 | 75.7 | 77.8 | 18 | 9 | NA | NA |
| Cho 2012 | Korea | III | 46.6 | 80 | 14.7 | 87.8 | 41 | 8 | 5 | 0 |
| Henaux 2012 | France | IV | 85.4 | 36.4 | 34 | 100 | 9 | 5 | 0 | NA |
| Mashhadinezhad 2012 | Iran | III | 31 | 73.3 | >9 | 100 | 15 | 2 | 2 | 0 |
| Kantelhardt 2012 | Germany | IV | NA | NA | NA | NA | 6 | NA | 1 | NA |
| Rizvi 2012 | Norway | III | >20 | NA | >6 | 87.5 | 20 | 7 | 7 (40)^1^ | 0 (40)^1^ |
| Aldrian 2012 | Austria | III | 59 | NA | >24 | 100 | 24 | 3 | NA | 1 (25)^1^ |
| Konieczny 2012 | Germany | IV | 47.8 | 61.5 | >6 | 84.6 | 13 | 3 | 3 | NA |
| Fan 2013 | Taiwan | IV | 45 | 66.7 | 24 | 100 | 24 | 4 | NA | 0 |
| Martirosyan 2013 | USA | III | 69 | 43.1 | 4.7 | 94.1 | 51 | 4 | NA | NA |
| Steltzlen 2013 | France | IV | 64.3 | 64.3 | 11 | 92.9 | 14 | 1 | 2 | 0 |

LE: Level of evidence. NU: Non-union. RO: Re-operation. I: Infection. NA: Not available.

^1^The data in brackets represent the real samples used for calculating corresponding rates.
