# Supplementary material for: Pooled Analysis of Non-Union, Re-Operation, Infection, and Approach Related Complications after Anterior Odontoid Screw Fixation
Source: PLoS One. 2014 Jul 24;9(7):e103065. doi: 10.1371/journal.pone.0103065 (PMC4109995; doi:10.1371/journal.pone.0103065)
Supplement: Table S2 — Characteristics of the studies included for analyzing approach related complications. (DOC) [file pone.0103065.s002.doc]

**Table S2.** Characteristics of the studies included for analyzing approach related complications.

| **Study** | **Country** | **LE** | **Age**  **(year)** | **Male%** | **Follow-up**  **(month)** | **Type Ⅱ%** | **D**  **(n)** | **H**  **(n)** | **E**  **(n)** | **W**  **(n)** | **S**  **(n)** |
| --- | --- | --- | --- | --- | --- | --- | --- | --- | --- | --- | --- |
| Geisler 1989 | USA | IV | 58 | 66.7 | >6 | 100 | NA | NA | NA | 0 | 0 |
| Etter 1991 | Switzerland | IV | 53.8 | 72.7 | >12 | 81.8 | NA | NA | NA | 2 | NA |
| Montesano 1991 | USA | IV | 57 | 71.4 | 24 | 100 | 2 | 1 | NA | 0 | 0 |
| Knoringer 1992 | Germany | IV | NA | NA | NA | NA | NA | NA | NA | 1 | NA |
| Chiba 1993 | Japan | IV | 38 | 81.2 | 22 | 77.8 | NA | NA | 1 | NA | NA |
| Chang 1994 | Taiwan | III | 42 | 83.3 | >12 | 100 | 2 | 3 | NA | 0 | 0 |
| Rainov 1996 | Germany | IV | 36 | 65.7 | >6 | 91.4 | NA | NA | NA | NA | 0 |
| Chiba 1996 | Japan | IV | NA | NA | >24 | 78.3 | NA | NA | NA | NA | 0 |
| Berlemann 1997 | Switzerland | IV | 75 | 52.6 | 30 | 100 | NA | NA | NA | 1 | 0 |
| Jenkins 1998 | USA | III | 59 | 57 | 8.5 | 100 | 2 | 1 | NA | NA | 0 |
| Henry 1999 | France | IV | 57 | 59.3 | 16.6 | 35.8 | 2 | NA | NA | NA | NA |
| Subach 1999 | USA | IV | 35 | 61.5 | 30 | 100 | 0 | 0 | NA | 0 | NA |
| ElSaghir 2000 | Germany | IV | 45 | 53 | 26 | 100 | 3 | NA | NA | NA | 0 |
| Apfelbaum 2000 | USA | III | 50.1 | 67 | 18.2 | 93.9 | NA | NA | 1 | NA | 1 |
| Alfieri 2001 | Italy | IV | >24 | 66.7 | NA | 100 | 1 | NA | NA | NA | 0 |
| Borm 2003 | Germany | III | 66.8 | 59.3 | 16.6 | 100 | NA | 1 | NA | NA | NA |
| Lee 2004 | Taiwan | IV | 37.2 | 77.1 | 14.6 | 81.3 | NA | NA | NA | NA | 0 |
| Fountas 2005 | USA | IV | 47.7 | 64 | >24 | NA | 0 | 0 | NA | 0 | 0 |
| Lee 2006 | Korea | IV | 43.7 | 80 | 18.6 | 90 | 1 | NA | NA | 0 | NA |
| Platzer 2007 | Austria | III | 54 | 46.4 | >24 | 100 | NA | NA | NA | NA | 1 |
| Ahmed 2007 | Saudi | IV | 35 | 83.3 | 10 | 100 | NA | NA | 1 | NA | NA |
| Chi 2007 | China | IV | 37.2 | 60 | 15.7 | 60 | 0 | 0 | 0 | 0 | 0 |
| Collins 2008 | UK | IV | 68.9 | 73.3 | 18.3 | 100 | 1 | NA | NA | NA | NA |
| Srinivasan 2008 | India | IV | >17 | NA | >2 | 100 | 4 | NA | 0 | NA | 0 |
| Sucu 2008 | Turkey | IV | 54.8 | 60 | 15.7 | 60 | 0 | 0 | 0 | NA | NA |
| Agrillo 2008 | Italy | IV | 73 | 66.7 | 8 | 100 | 2 | NA | NA | NA | NA |
| Koller 2009 | Austria | IV | 57.9 | 90.9 | 72.8 | 90.9 | 1 | NA | NA | NA | NA |
| Dailey 2010 | USA | IV | 81.2 | 47 | 15 | 94.7 | 20 | NA | NA | NA | NA |
| Osti 2011 | Austria | III | 79.6 | 55 | 67.2 | 100 | NA | NA | NA | NA | 0 |
| Yang 2011 | China | III | 35 | 69 | 13.5 | 82.8 | NA | 1 | NA | 1 | 0 |
| Wang 2011 | China | II | 47.1 | 61.9 | 25.1 | 82.2 | 2 | NA | 0 | NA | 0 |
| Mayer 2011 | Austria | III | 78.1 | 72.2 | 75.7 | 77.8 | NA | NA | NA | NA | 0 |
| Grossberg 2011 | USA | IV | 71.4 | 57.9 | NA | 100 | 6 | 1 | NA | NA | NA |
| Mashhadinezhad 2012 | Iran | III | 31 | 73.3 | >9 | 100 | NA | NA | NA | NA | 0 |
| Rizvi 2012 | Norway | III | >20 | NA | >6 | 87.5 | NA | NA | NA | 0 | NA |
| Fan 2013 | Taiwan | IV | 45 | 66.7 | 24 | 100 | NA | 0 | NA | NA | 0 |
| Martirosyan 2013 | USA | III | 69 | 43.1 | 4.7 | 94.1 | 17 | NA | NA | NA | NA |

LE: Level of evidence. D: Dysphagia. H: Hoarseness. R: esophageal /retropharyngeal injury. W: Wound hematomas. S: Spinal cord injury. NA: Not available.
